# Characteristics of Emergency Medicine Specimen Bank Participants Compared to the Overall Emergency Department Population

**DOI:** 10.5811/westjem.2022.11.57981

**Published:** 2023-02-25

**Authors:** Alexis Vest, Brandon Sonn, Richie Puls, Cosby Arnold, Zach Devney, Arwah Ahmed, Olivia Pallisard, Andrew A. Monte

**Affiliations:** * University of Colorado School of Medicine, Department of Emergency Medicine, Aurora, Colorado; † University of Colorado School of Medicine, Center for Bioinformatics & Personalized Medicine, Aurora, Colorado; ‡ University of Colorado, Skaggs School of Pharmacy and Pharmaceutical Sciences, Aurora, Colorado; § Denver Health and Hospital Authority, Rocky Mountain Poison & Drug Center, Denver, Colorado

## Abstract

**Introduction:**

Biorepositories lack diversity both demographically and with regard to the clinical complaints of patients enrolled. The Emergency Medicine Specimen Bank (EMSB) seeks to enroll a diverse cohort of patients for discovery research in acute care conditions. Our objective in this study was to determine the differences in demographics and clinical complaints between participants in the EMSB and the overall emergency department (ED) population.

**Methods:**

This was a retrospective analysis of participants of the EMSB and the entire UCHealth at University of Colorado Anschutz Medical Center (UCHealth AMC) ED population across three periods: peri-EMSB; post-EMSB; and COVID-19. We compared patients consented to the EMSB to the entire ED population to determine differences in age, gender, ethnicity, race, clinical complaints, and severity of illness. We used chi-square tests to compare categorical variables and the Elixhauser Comorbidity Index to determine differences in the severity of illness between the groups.

**Results:**

Between February 5, 2018–January 29, 2022, there were 141,670 consented encounters in the EMSB, representing 40,740 unique patients and over 13,000 blood samples collected. In that same time, the ED saw approximately 188,402 unique patients for 387,590 encounters. The EMSB had significantly higher rates of participation from the following: patients 18–59 years old (80.3% vs 77.7%); White patients (52.3% vs 47.8%), and women (54.8% vs 51.1%) compared to the overall ED population. The EMSB had lower rates of participation from patients ≥70 years, Hispanic patients, Asian patients, and men. The EMSB population had higher mean comorbidity scores. During the six months after Colorado’s first COVID-19 case, the rate of consented patients and samples collected increased. The odds of consent during the COVID-19 study period were 1.32 (95% CI 1.26–1.39), and the odds of sample capture were 2.19 (95% CI 2.0–2.41).

**Conclusion:**

The EMSB is representative of the overall ED population for most demographics and clinical complaints.

## INTRODUCTION

Personalized medicine can improve the care of patients with acute conditions.^1^ Patients with genotype data may have treatments changed in the emergency department (ED) for conditions such as myocardial infarction and respiratory failure. For instance, clopidogrel is not recommended in one-third of myocardial infarction patients who are *CYP2C19*-poor metabolizers due to the risk of stent thrombosis,^2^ and succinylcholine should be avoided when a patient has a variant in *RYR1*, which predisposes to malignant hyperthermia.^3^ However, there remains a shortage of data from patient populations with diverse ancestral backgrounds and acute care diagnoses needed to push discovery studies in acute care. Genome-wide association studies typically require 1,000 patients with a phenotype and 1,000 patients without to be adequately powered. There are not large cohorts with diverse ancestral backgrounds and a broad spectrum of clinical diseases to power acute-care personalized medicine studies.^4^

The largest biobanks in the United States (US) consist of primarily non-Hispanic White participants. For example, the Marshfield Medical Clinic biobank, the largest general biobank in the US, is composed of 98% non-Hispanic White participants,^5^ and the Geisinger Biobank is composed of greater than 95% non-Hispanic White individuals.^6^ The Vanderbilt University Medical Center BioVU biobank has slightly better diversity, with 75% of participants being non-Hispanic White.^7^ While these demographics are representative of the populations surrounding the biobanks, their applicability to acute clinical situations is limited because they are not representative of the demographics typically cared for in EDs.^8^ The *All of Us* program is the most diverse genomic enrollment biobank to date, although acute clinical data is not currently available through the program.^9^ Inclusion of ancestrally diverse groups allows for capture of rare genetic variants that can cause discordant clinical responses in underrepresented minority groups.^10^ Lack of diversity can limit the clinical applicability of findings resulting from the biobank data and can worsen the health inequities for minority groups seeking acute clinical care in EDs.

Emergency departments represent an untapped resource of ancestral and phenotypically diverse cohorts due to their increased demographic diversity and variety of acute health conditions encountered and treated, as compared to other clinic sites. In 2018, US ED visits were comprised of 53.1% non-Hispanic White, 26.5% non-Hispanic Black, 16.5% Hispanic (15.2% Hispanic-White, 0.9% Hispanic-Black, and 0.4% Hispanic-other).^11^ Additionally, EDs across the nation diagnosed and treated almost 50,000 distinct health problems across 150 million patient visits. The variety of clinical diseases and drugs administered provide endless potential for personalized medicine discovery. The ED is a unique and ideal location for personalized medicine research to improve the care for a wide variety of patients and clinical conditions. However, the coronavirus disease 2019 (COVID-19) pandemic altered the demographics of patients presenting to EDs^12^ and affected their willingness to participate in research.^13^ Thus, we believe that examination of this potential confounding factor is necessary to interpret how research populations compare to overall clinical populations.

The Emergency Medicine Specimen Bank (EMSB) at the University of Colorado is the first large-scale biobank that seeks to enroll all patients in an acute care setting.^14^ The EMSB facilitates research studies by pairing clinical data with biologic samples in a group of patients with acute illness with a broad range of clinical severity.^14^ Our overall objective in this study was to compare the demographics and clinical conditions of those enrolled in the EMSB, accounting for how the COVID-19 pandemic affected representation, compared to the overall ED population from which the cohort was drawn.

**Population Health Research Capsule**

What do we already know about this issue?

*The lack of ancestral and clinical diversity in biobanks can cause rare genetic variants to go unidentified, limiting the applicability of precision medicine in acute care conditions*.

What was the research question?

*Does the Emergency Medicine Specimen Bank (EMSB) reflect the diverse patient population in the ED?*

What was the major finding of the study?

*The EMSB enrolled fewer older Hispanic and Asian patients compared to the overall ED population (P-value<0.001)*.

How does this improve population health?

*Non-English speaking patients are enrolled at a lower rate, although all clinical complaints are represented in acute care biorepositories*.

## METHOD

### Clinical Setting and Patient Population

The EMSB is housed at the University of Colorado Hospital ED Anschutz Medical Campus (UC-AMC). The ED at this UC-AMC is a large-volume academic facility with approximately 100,000 visits annually, although in 2018 at the time the EMSB was initiated, volume was approximately 80,000 visits per year. UC-AMC is in Aurora, CO, adjacent to Denver, and is the second-largest city in the state.

### Inclusion Criteria/Exclusion Criteria

The EMSB was initiated in the UC-AMC ED on February 5, 2018. Patients eligible for the EMSB include those presenting to UC-AMC who are >17 years of age, speak English or Spanish, and are medically stable to consent or have a medical durable power of attorney (MDPOA). The EMSB researchers and trained clinical staff approach all eligible patients for consent to participate in this biobank program. All patients who have an intravenous line (IV) placed as part of their routine care have a blood sample collected, and the EMSB keeps samples from consented participants. Consent, sample collection, sample sorting, and sample processing occur in the ED.

The inclusion and exclusion criteria are outlined in the electronic health record (EHR) system used by UCHealth (Epic Systems Corporation, Verona, WI). Patients are excluded if their clinical condition precludes the ability to consent, and there is no MDPOA available. The consent lasts for a year after signing, allowing for collection of samples and clinical data from subsequent ED visits without additional consent.

### Waiver of Consent and Institutional Review Board Approval

Obtaining traditional informed consent prior to sample collection is not feasible for all ED subjects because of the nature of the ED clinical interaction. To overcome this barrier, the EMSB operates under a temporary waiver of consent approval status,^14^ which allows for collection of the blood samples during routine clinical draws, although the samples are only kept for research when matched with a consent, which occurs later in the ED visit. This protocol was approved by the Colorado Multiple Institutional Review Board and adheres to the ethical principles for medical research outlined in the Declaration of Helsinki.

### Data Extraction

We examined three study periods: the peri-EMSB, January 12, 2017–January 13, 2019; the post-EMSB, February 5, 2018–January 22, 2022; and the COVID-19 era.

### Peri-EMSB

Within the established time frame, our goal was to allow for examination of EMSB inclusion as compared to the overall ED population including detailed data on visit diagnoses and medications administered. This also allowed examination of the impact of EMSB implementation over time. We used our data warehouse, Health Data Compass, for data extraction. This de-identified dataset included detailed records of all *International Classification of Diseases, 10^th^ Revision* (ICD-10) codes, chief complaints, and medication administrations, as well as basic demographic information such as age, race, ethnicity, and gender for all patients presenting to the ED.

#### Post-EMSB

As with the peri-EMSB period, this time frame allowed for examination of the demographics and clinical presentation of EMSB populations compared to the overall ED population. This data extraction allowed for examination of detailed clinical variables with total consent rates across the ED and the EMSB population from the inception of the EMSB on February 5, 2018, to the most recent data extraction on January 22, 2022. We used data collected under the EMSB protocol. The EMSB collects a limited dataset from all ED patients for preliminary hypothesis exploration but does not collect the detailed data obtained for the peri-EMSB cohort. The post-EMSB data allows for examination of changes over a longer time period and is more flexible than the peri-EMSB dataset. This data includes demographics (age, gender, race, and ethnicity), chief complaint, diagnosing *International Classification of Diseases, 10^th^ Rev* (ICD-10) code, and time of sample. All clinical data available in the EHR can be extracted for EMSB consented patients under new specified ethics board-approved research protocols.

#### COVID-19

The COVID-19 pandemic had a major impact on who accessed healthcare and how healthcare was accessed. It also had an impact on the number of research staff who were available to enroll patients. Therefore, we examined enrollment specifically six months before (pre-COVID-19, September 1, 2019– February 29, 2020) and after the COVID-19 pandemic began (post-COVID, March 1–August 31, 2020). During this study period, we examined consent rates, sample collection rates, and the rate of patients approached for consent.

### Statistical Analyses

The unit of analysis was the ED visit. We made comparisons between categorical variables using chi-square tests. Comorbidity scores were calculated using ICD-10 codes and the Elixhauser Comorbidity Index score tool.^9^ We used ANOVA testing to compare Elixhauser scores between the two groups. We calculated the EMSB approach rate as the number of consented patients plus the number of declined patients/number total patients in the ED. We then calculated consent rates as the number of consents/number approached patients. The sample collection rate was calculated as the number samples collected/number encounters involving consented patients. Each of these rates was calculated for each month in their respective time periods. We compared mean rates across study periods using ANOVA and odds ratios.

## RESULTS

### Peri-EMSB Study Period

In the peri-EMSB study period (January 12, 2017–January 13, 2019), there were 119,450 visits in the overall ED population and 7,120 visits consented to the EMSB. The proportion of White and Black patients was higher in the EMSB population compared to the overall ED population (Table 1). The greater representation of Blacks was primarily driven by Black men who were less likely to participate in the EMSB program (9.7% EMSB participant vs 10.3% overall ED population). There was a lower representation of Asians in the EMSB in comparison to the overall ED population, and this trend was also consistent across the post-EMSB study period (see below). The EMSB enrolled fewer patients >70 years in the peri-EMSB period, and this continued across all study periods (Table 2).

**Figure 1:**
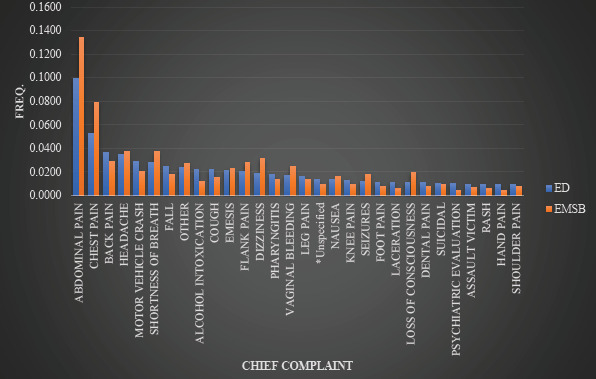
Frequencies of the most common chief complaints for emergency department and Emergency Medicine Specimen Bank (EMSB) encounters during the peri-EMSB study period.

During the peri-EMSB study period, the chief complaints of the EMSB consented cohort were consistent with the overall ED population (Figure 1); abdominal pain (13% vs 10%) and chest pain (8% vs. 5%) were the most common in both the EMSB and the overall ED cohorts, respectively. Of the 50 most common chief complaints, 45 were shared across the groups. During this study period there were a total of 773,652 individual ICD-10 codes represented for 342,534 encounters. The ICD-10 codes were similar across the overall ED population and the EMSB consented groups; six of the 20 most common ICD codes were found in both the ED and the EMSB (Table 3). Patient encounters that were consented to the EMSB had higher Elixhauser comorbidity scores compared to the overall ED population (0.692 vs 0.262, respectively). The EMSB-consented encounters had Elixhauser scores ranging from −18 to 39 (median 0, IQR 1,0) while patients without EMSB-consented encountered had scores ranging from −19 to 39 (median 0, IQR −1,0).

### Post-EMSB Study Period

During the post-EMSB study period (February 5, 2018–January 22, 2022), the UC-AMC saw 188,402 patients for 387, 590 encounters (Table 1). This population consisted of 47.8% White patients, over half were <60 years (78.2%), and a little over a quarter of patients were Hispanic (27.33%). These visits had a total of 38,127 diagnoses codes for 778 chief complaints. The median age was 40 years (interquartile range [IQR] 29, 57) in the EMSB and 40 years (IQR 28, 57) in the general ED population. The proportion of EMSB participants aged 18–59 years were higher than the proportion of ED patients in the same age range (Table 2); the greatest number of patients seen in the ED and the greatest proportion of consented EMSB participants were in the 18- to 29-year-old range. There were, however, lower rates of consent in patients aged 70–81+ years. Only subjects within the 60–69 years age bracket had similar representation compared to the general ED population. Only about 9% of all EMSB consents come from patients ≥70.

**Figure 2:**
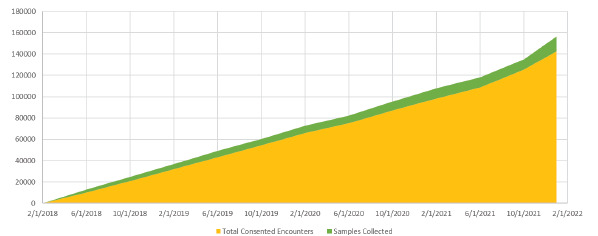
Samples collected for consented encounters increased at generally the same rate as the number of consented encounters. The frequency of consented encounters is found on the left vertical axis including a total of ~140,000 consented encounters over the first four years of the Emergency Medicine Specimen Bank program. The number of total samples collected was ~14,000 collected over this study period.

**Figure 3:**
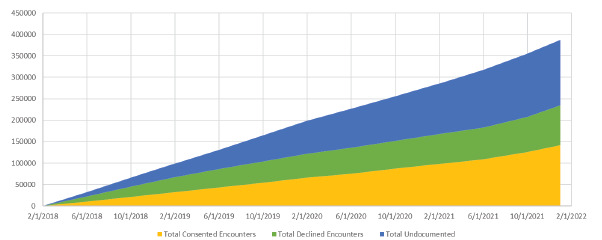
Rate of encounter consent, decline, and lack of documentation for Emergency Medicine Specimen Bank program. Over time, the number of all encounter types has increased. A steady increase in the number of consented encounters, increased rate of undocumented encounters, and decreased rate of declined encounters has been observed. The total number of emergency department visits over this period was ~300,000 encounters.

The number of samples collected increased proportionally to the number of consented encounters (Figure 2) with >14,000 samples collected. The number of EMSB consents increased steadily over the study period; 36.7% of all patients presenting to the ED were consented to the EMSB at the end of the three study periods (Figure 3). The number of samples collected is lower than the number of consented encounters because samples are only drawn when a subject has an IV placed for clinical care; over the post-EMSB study period only 59.8% of EMSB consented visits had an IV placed.

Over the post-EMSB study period, the proportion of patients who declined to participate in the EMSB steadily decreased from a peak of about 36% of all ED patients in August 2018 to only 23.7% as of January 22, 2022 (Figure 3). The number of undocumented encounters that did not receive a consent or a decline documented increased from 34% in August 2018 to 39.6% on January 22, 2022. The proportion of females presenting to the ED for care was slightly higher than males, but a higher percentage of females were consented when compared to males (51.7% vs 56.7%).

The rate of participation in the EMSB for subjects of Hispanic ethnicity differed from that of the general ED population (23.3% vs 28.0%). This difference seemed to be primarily driven by Hispanic males; there was a lower rate of consent in male Hispanic patients in the EMSB program compared with the overall ED population (23.0% vs 26.3%). On the other hand, there was no significant difference in rate of participation compared with overall ED population for Hispanic females (27.9% vs 27.6%).

There were 13 languages in 10,231 non-English or Spanish-speaking encounters. The limited language availability of the EMSB consent form greatly influenced the underrepresentation of Asians. Only 54% of Asian ED patients spoke English making 46% of patients ineligible due to language alone.

### COVID-19 Pandemic

**Figure 4:**
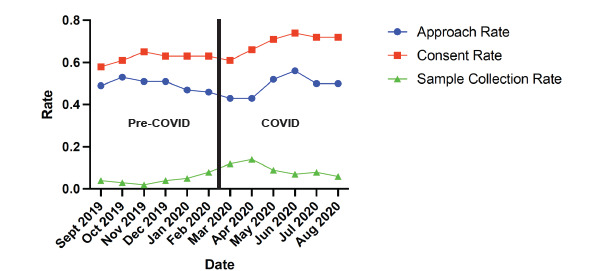
Consent, sample collection, and patient approach rates by month during the COVID-19 study period.

In the stx months before the pandemic (pre-COVID), there were 44,113 ED visits by 36,182 patients with 16,934 patients approached (46.80%), There was a total of 10,431 consented patients (61.60%), and 748 specimens collected. In the six months after the COVID-19 pandemic began (post-COVID), there were 36,228 ED visits with 29,768 patients seen, 13,911 patients approached (46.73%), 9,457 consented patients (67.98%), and 1,371 samples collected. There was no difference in the approach rate before or after the pandemic began (pre-COVID mean rate 0.47; COVID mean rate 0.47). The consent rate was higher in the COVID-19 period (pre-COVID mean rate 0.62; post-COVID mean rate 0.68) (Figure 4). The sample collection rate also increased in the post-COVID study period (pre-COVID mean rate 0.07; post-COVID mean rate 0.14). The odds of consent during the post-COVID study period were 1.32 (95% CI 1.26–1.39), and the odds of sample capture were 2.19 (95% CI 2.0–2.41).

## DISCUSSION

The EMSB has increased enrollment and sample collection through integration into the standard clinical workflow. The 40,740 visits consented to this biorepository are largely representative of the ~188,400 ED patients, complaints, and diagnoses seen over the enrollment period. Additionally, the EMSB collected more than 14,000 whole blood samples from these patients over the same time from these subjects seen for emergent care. This patient and complaint diversity will allow for personalized medicine discovery studies that are already underway. This broad enrollment strategy has allowed the EMSB to provide clinical data and biologic samples for numerous studies including stroke, anti-emetic effectiveness, and COVID-19.^15^,^16^

While the age of the EMSB population is largely representative of the overall ED population, there was higher representation of younger participants. This was not unexpected as previous studies have described increased willingness of younger patients to participate in research.^17^,^18^ We hypothesize that younger patients may have higher support for personalized medicine research and may be more willing to participate due to comfort with digital consent platforms.^19^ There are fewer older participants consented to the EMSB, possibly due to increased frequency of advancing medical conditions.^18^ When conditions such as hearing loss, vision loss, or dementia are present, this can increase the burden on staff in an informed consent process; therefore, fewer older subjects may be approached to participate.^19^ Also, with increasing health concerns, older subjects may be unwilling to put themselves at additional perceived risk of participating in a research program.^17^,^20^ We will address the age-based disparity in consent and participation within the EMSB moving forward with targeted enrollment strategies.

There is greater representation of women within the consented EMSB cohort, similar to other biobank programs, but varying from prior epidemiologic studies that demonstrate females, especially over the age of 50, have lower rates of participation in clinical trials and research follow-up.^17^,^18^ On a global scale, there is greater support among women for personalized medicine research and biobanking programs compared to men,^19^ which is supported by our data.

The EMSB participants have, on average, a higher comorbidity score than the overall ED population. While our analyses demonstrate that chief complaints are similar between EMSB consented and the overall ED population, consented patients may be more likely to have comorbid disease. This is likely because patients with more complex medical histories have longer ED stays and are more likely to have repeat visits and blood draws. These factors increase the opportunity for research staff to obtain consent for the biobank.

Our demographic data demonstrates systematic exclusion of some groups. Patients who are unconscious, are unable to consent due to their condition, or do not speak English or Spanish are not consented to the EMSB. While consent for one year after the index visit allows for capture of some subsequent visit data and samples, critically ill patients with only one visit are underrepresented in the EMSB. This may limit our ability to rapidly advance personalized medicine in some conditions. Furthermore, ED patients spoke 13 languages other than English and Spanish. These patients were also systematically excluded by the nature of the consent process. Over 10,000 patients were ineligible over the study period due to language exclusion, and this may have led to failure to capture rare genetic variants with high frequency in non-English/Spanish speaking ancestral populations. Translation into additional languages or utilization of interpreters could allow inclusion of these patients in the future, although that process may be too challenging for patients and research staff in this self-consent model. This research can be considered minimal risk, given that the data and samples are combined into large datasets and de-identified prior to analyses. This raises the question of whether consent is necessary for this design, given the implications for systematic exclusion of some demographic groups.

The COVID-19 pandemic altered the EMSB consent and sample collection processes. As of March 16, 2020, researchers without clinical responsibilities, including students and interns previously aiding in enrollment and prompting sample collection, were forced to work remotely to minimize their risk of contagion. This impeded the ability to consent patients in the ED or work with clinical staff for sample collection. Additionally, many new hospital processes and protocols were implemented to protect the clinic staff from illness. This resulted in fewer potential subjects being approached to participate in the biobank program, thereby increasing undocumented encounters and prompting us to adjust our consent and sample collection workflow. Subsequently, the number of consents has increased, averaging around 50% of monthly ED encounters over the past year. Additionally, while subjects can sign a one-year consent, the number of consented encounters has risen, but without EMSB researchers on site to remind clinical staff to collect samples, the percent of samples collected compared with consented visits has declined. Despite this, it is encouraging that the proportion of declined encounters has steadily decreased since inception. The consent rate and sample collection rates increased significantly during the COVID-19 pandemic compared to the six months prior. This was likely due to increased patient and clinician interest in research paired with operational improvements to ease consent and sample collection.

## LIMITATIONS

The English/Spanish language eligibility criteria particularly limited Asian recruitment in our ED; less than half of all Asians who were seen spoke English. Visits in which the patient was discharged or admitted quickly provided less time for patient consent. Even if consented, not all clinical complaints were well represented with a blood sample since many musculoskeletal injuries do not require an IV and thus don’t provide a biologic sample. This may have limited our ability to capture genetic variants associated with analgesic effectiveness, for example. The EMSB cohort is biased toward including more severe clinical complaints that require longer work-up time in the ED. Also, while the EMSB aims to increase diversity and be representative of the ED patient population, the cohort is not entirely representative of the Denver area.

The population treated at the UC-AMC ED is still a majority White, although not as high as the Denver population (52.4% in UC-AMC ED, 80.9% in Denver County), and Blacks have greater representation (21.3% in UC-AMC ED, 9.8% in Denver County).^21^ The location of the hospital may have contributed to this over-representation of Blacks, and in fact, increased the diversity in our enrollment.^4^ Enrollment and sample capture processes have changed over time. Initially, there was excitement about the project, which led to high enrollment rates. Enrollment fell in the latter half of the first year of implementation. Providing increased education on the protocol and sharing study results with the clinical staff have been associated with increased enrollment rates in subsequent years.

## CONCLUSION

The Emergency Medicine Specimen Bank is representative of the overall ED population for most demographics and clinical complaints. While barriers to inclusion remain, integration into clinical workflow was associated with increased consent and sample collection numbers. Enrollment in EDs can increase the diversity of patients and clinical conditions represented in biobanks.

## Figures and Tables

**Table 1:** Demographics of study population across study periods. Only unique medical record numbers counted for the demographics.

Demographic variable	Peri-EMSB		Post-EMSB		COVID-19 Period	
		
	EMSB consented N= 7,120	Overall ED population N=119,450	EMSB consented N=40,740	Overall ED population N=188,402	EMSB consented N=15,139	Overall ED population N= 59,251
Median age (IQR)	43 (28,56)	41 (27,55)	40 (29,57)	40 (28,57)	39 (28,55)	41 (28,57)
Male gender (%)	41.5	42.1	45.2	48.5	42.4	47.8
Race (%)						
American Indian or Alaskan Native	0.3	0.42	0.9	0.7	1.0	0.8
Asian	1.4	2.7	1.9	3.3	1.8	3.5
Black	22.2	20.2	20.9	20.6	26.4	23.6
Native Hawaiian or other Pacific Islander	0.2	0.29	0.2	0.2	0.5	0.4
White	50.6	48.0	52.3	47.8	46.6	45.1
More than one race	4.5	3.8	0.9	0.7	1.0	0.8
Other	19.1	21.9	22.4	24.5	22.4	24.6
Patient refused, or unknown	0.2	2.7	0.1	1.5	0.3	1.3
Hispanic ethnicity (%)	23.4	24.8	27.3	27.3	27.6	27.6

*ED*, emergency department; *EMSB*, Emergency Medicine Specimen Bank; *COVID-19*, coronavirus disease 2019; *IQR*, interquartile range.

**Table 2:** Age distribution of EMSB consents during the peri-EMSB (January 12, 2017–January 13, 2019), Post-EMSB (February 5, 2018–January 22, 2022), and COVID-19 (September 01,2019 – August 31, 2020) study periods.

Age range	Peri-EMSB		Post-EMSB		COVID-19 Period	
		
	EMSB population N=40,740	Overall ED population N=188,402	EMSB population N= 7,120	Overall ED population N=119,450	EMSB population N=15,139	Overall ED population N= 59,251
18–29	27.3%	25.1%	26.9%	25.0%	27.3%	25.7%
30–39	22.0%	20.6%	21.7%	20.6%	21.9%	21.3%
40–49	16.2%	15.5%	15.9%	15.3%	16.2%	16.1%
50–59	14.9%	14.0%	14.8%	13.8%	14.9%	14.4%
60–69	10.8%	11.0%	11.1%	11.1%	10.5%	11.2%
70–79	5.8%	6.7%	6.3%	6.8%	6.0%	6.8%
80+	2.8%	4.2%	3.2%	4.3%	3.1%	4.4%

*EMSB*, Emergency Medicine Specimen Bank; *ED*, emergency department; *COVID-19*, coronavirus disease 2019.

**Table 3:** Frequency of top 20 International Classification of Diseases, 10th Revision diagnosis codes in the peri-EMSB study period.

ICD-10 Codes	Overall ED population, N = 159,899 (%)	EMSB Consented, n= 7,871 (%)
I10 Essential hypertension	21.0	21.2
F17.210 Nicotine dependence, cigarettes, uncomplicated	20.0	18.9
E11.9 Type 2 diabetes, without complication	8.8	13.5
F17.200 Nicotine dependence, uncomplicated	6.5	8.1
F41.9 Anxiety disorder, unspecified	3.3	6.4
M54.5 Low back pain	3.2	6.4
G89.29 Other chronic pain	3.2	6.0
J44.9 Chronic obstructive pulmonary disease	3.1	5.5
J45.909 Unspecified asthma, uncomplicated	2.9	5.1
M54.9 Dorsalgia, unspecified	2.9	5.1
I25.10 Atherosclerotic heart disease of native coronary artery without angina pectoris	2.4	4.2
F10.920 Alcohol use, unspecified with intoxication, uncomplicated	2.3	4.1
M54.2 Cervicalgia	2.2	3.9
J06.9 Acute upper respiratory infection, unspecified	2.2	3.8
J02.9 Acute pharyngitis, unspecified	2.0	3.6
F32.9 Major depressive disorder, single episode, unspecified	1.8	3.1
F10.129 Alcohol abuse with intoxication, unspecified	1.7	3.0
F10.120 Alcohol abuse with intoxication, uncomplicated	1.7	3.0
I50.9 Heart failure, unspecified	1.6	2.9
E78.5 Hyperlipidemia, unspecified	1.6	2.8

*EMSB*, Emergency Medicine Specimen Bank; *ICD*, International Classification of Diseases, 10th Rev.
